# Supplementary material for: Follistatin-like 1 and Biomarkers of Neutrophil Activation Are Associated with Poor Short-Term Outcome after Lung Transplantation on VA-ECMO
Source: Biology (Basel). 2022 Oct 8;11(10):1475. doi: 10.3390/biology11101475 (PMC9598172; doi:10.3390/biology11101475)
Supplement: Supplementary file 1 [file biology-11-01475-s001.zip › biology-1838459-supplementary.pdf]

Supplementary material

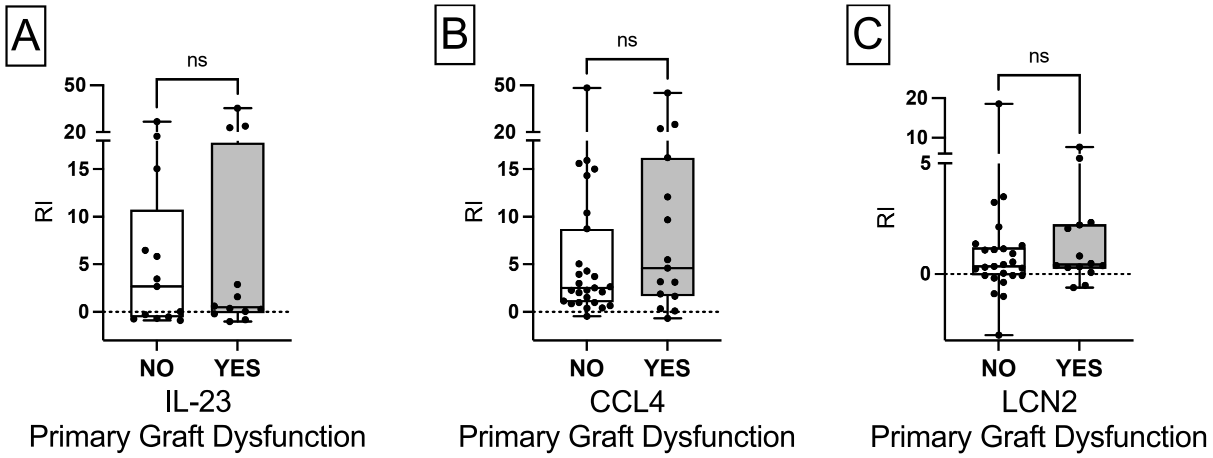

Supplementary Figure S1: There is no statistically significant difference in RI of IL-23, CCL4 and LCN2 serum concentrations between patients diagnosed with PGD and patients without PGD (**A–C**). CCL4, chemokine 4; LCN2, IL-23, interleukin-23; lipocalin 2; ns, not significant; RI relative increase.
